# A Review of the Mechanism of Antagonism of N-methyl-D-aspartate Receptor by Ketamine in Treatment-resistant Depression

**DOI:** 10.7759/cureus.2652

**Published:** 2018-05-18

**Authors:** Yasar Sattar, John Wilson, Ali M Khan, Mahwish Adnan, Daniel Azzopardi Larios, Shristi Shrestha, Quazi Rahman, Zeeshan Mansuri, Ali Hassan, Nirav B Patel, Nargis Tariq, Sharaad Latchana, Stefany C Lopez Pantoja, Sadiasept Vargas, Naveed A Shaikh, Fawaduzzaman Syed, Daaman Mittal, Fatima Rumesa

**Affiliations:** 1 Research Assistant, Kings County Hospital Center, New York, USA; 2 Adult Psychiatry, SUNY Downstate Medical Center; 3 Psychiatry Resident, University of Texas Rio Grande Valley, Harlingen, Texas, USA; 4 Center for Addiction and Mental Health, University of Toronto, toronto, CAN; 5 Child Psychiatry, Kings County Hospital Center; 6 MPH, State University of New York; 7 Child Pscyhiatry, SUNY Downstate Medical Center; 8 Psychiatry, Texas Tech University Health Sciences Center at Odessa/permian Basin; 9 Medical Graduate, American University of Antigua; 10 Department of Medicine, Lasante Health; 11 Graduate, Avalon University School of Medicine; 12 Medical Student, American University of Integrative Sciences; 13 Graduate, Pontifical Catholic University of Ecuador, Chagrin Falls, USA; 14 Department of Medicine, Instituto Tecnológico De Santo Domingo, Santo Domingo, DOM; 15 Psychiatry, Kings County Hospital Center, Brooklyn, USA; 16 Internal Medicine, Sindh Medical College, Dow University of Health Sciences, Chicago, USA; 17 Pediatrics, Punjab Institute of Medical Sciences, ludhiana, IND; 18 Psychiatry, Icahn Elmhurst Hospital, New York, USA

**Keywords:** ketamine, nmda antagonist, suicide, nmda receptor, depression

## Abstract

The biochemical processes involved in depression go beyond serotonin, norepinephrine, and dopamine. The N-methyl-D-aspartate (NMDA) receptor has a major role in the neurophysiology of depression. Ketamine, one of the prototypical NMDA antagonists, works rapidly in controlling depressive symptoms, including acutely suicidal behavior, by just a single injection. Ketamine may rapidly increase the glutamate levels and lead to structural neuronal changes. Increased neuronal dendritic growth may contribute to synaptogenesis and an increase in brain-derived neurotrophic factor (BDNF). Activation of the mechanistic target of rapamycin (mTOR), as well as increased levels of BDNF, may increase long-term potentiation and result in an improvement in the symptoms of depression. The mechanisms of ketamine’s proposed effect as an off-label treatment for resistant depression are outlined in this paper.

## Introduction and background

According to the World Health Organization, suicide ranks among the top three causes of death worldwide amongst people aged 15 to 44 years and, in the year 2000, there was one death every 40 seconds worldwide due to suicide [1]. The Centers for Disease Control has summarized the facts about suicide for the United States (US) population, stating that suicide results in an estimated $51 billion in combined medical and work loss costs [2]. There are varieties of different risk factors for suicide, but mental illness is still the leading cause of suicide in the US [3]. Depression is one of the more prevalent mood disorders, affecting more than 300 million people worldwide, sometimes leading to a deadly fate in suicide [4]. With constantly evolving cutting-edge research on depression, investigators found that ketamine, the drug initially approved by the US Food and Drug Administration (FDA) as a general anesthetic agent in the 1970s, is clinically effective in treatment-resistant depression with suicidal behavior. Ketamine is an N-methyl-D-aspartate (NMDA) receptor antagonist that rapidly reduces suicidal ideations and depressive symptoms. Ketamine in any form, including oral, intravenous, and even intranasal routes, has been shown to reduce suicidal behavior in depressive patients quickly [5-7]. The focus of this paper is to simplify the diverse molecular mechanisms of ketamine that may provide a rapid response to depression and suicidal ideation.

The antidepressant and anti-suicidal actions of ketamine are possibly due to its effect on neurotransmitter levels, neuroinflammatory markers, and neurotrophic factors that lead to synaptogenesis and intracellular signaling mechanisms. Most of the pathways affected by ketamine are in the limbic system and involve the prefrontal cortex and hippocampus [8]. Animal and human model studies have led to a variety of mechanisms being hypothesized. Ketamine may increase glutamate levels [9], reduce the levels of neuroinflammatory chemicals such as interleukin-1 and interleukin-6 [10] and inhibit glycogen synthase kinase-3 [11]. It also stimulates synapse formation through activation of the mechanistic target of rapamycin (mTOR) [12] and up-regulates neurotrophic factors such as brain-derived neurotrophic factor (BDNF) [13] and eukaryotic elongation factor-2 [14].

Unfortunately, ketamine, like other NMDA antagonists, has not yet been approved for treatment-resistant depression in the US by the FDA. More studies should be done on NMDA and BDNF in the future to develop a treatment for depression that can effectively and efficiently control the depressive episode and provide baseline mood maintenance in the long-term.

Method

A total of 150 studies were included in our review by searching the keyword ‘ketamine’ and cross-referencing it with depression, suicide, NMDA antagonist, and mechanism of action of ketamine in suicide, in both the PubMed search index and Google Scholar for the years 2007 to 2017. This literature included studies involving peer review, systematic review, case series, case reports, and randomized clinical trials on depression with suicidal behavior in human and animal models. The material reviewed had to include a depiction of the mechanism of action of ketamine in depression and its connection to the NMDA receptor.

## Review

**Figure 1:**
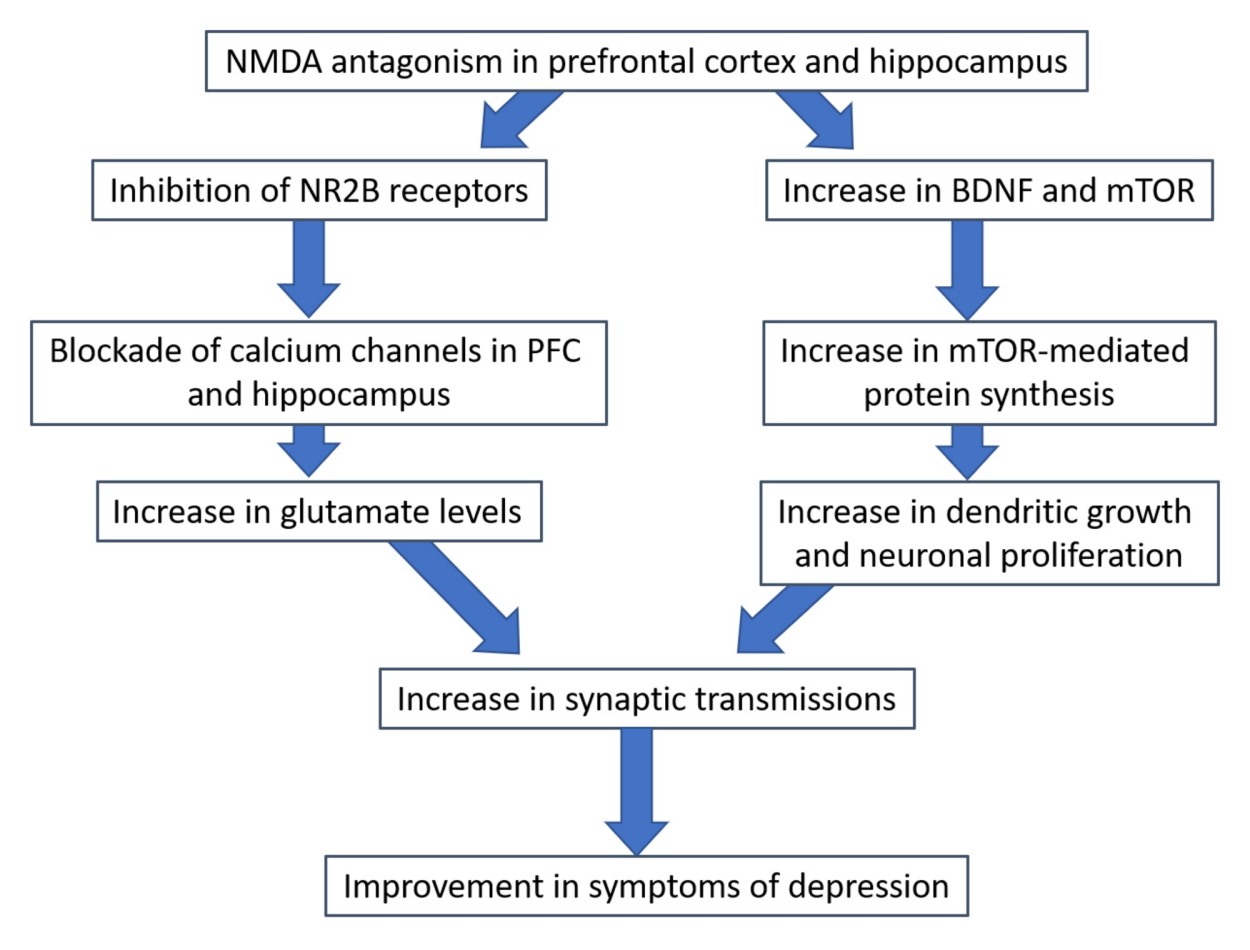
Proposed effect of NMDA antagonism on neurotransmission Abbreviations: NMDA, N-methyl-D-aspartate; BDNF, brain-derived neurotrophic factor; mTOR, mechanistic target of rapamycin; PFC, prefrontal cortex.

Our understanding of depression psychopharmacology needs no longer be limited to the serotonin receptor pathway. Recently, investigators have found multiple pathways involved in the neurophysiology of depression, including the NMDA and opioid receptor pathways. Ketamine, a product of phencyclidine, is effective in treating depression in a large number of studies [15-17]. Our discussion is entirely focused on the mechanism of action of its antidepressant effect, which starts at the NMDA receptor binding site and cascades through deep intracellular signaling to affect neuronal synaptic anatomic linkage. Ketamine’s antidepressant mechanism of action can be divided into two broad categories (Figure 1): neurotransmitter changes, and intracellular signaling/neurotrophic factor modulation.

Ketamine action at the neurotransmitter level

**Figure 2:**
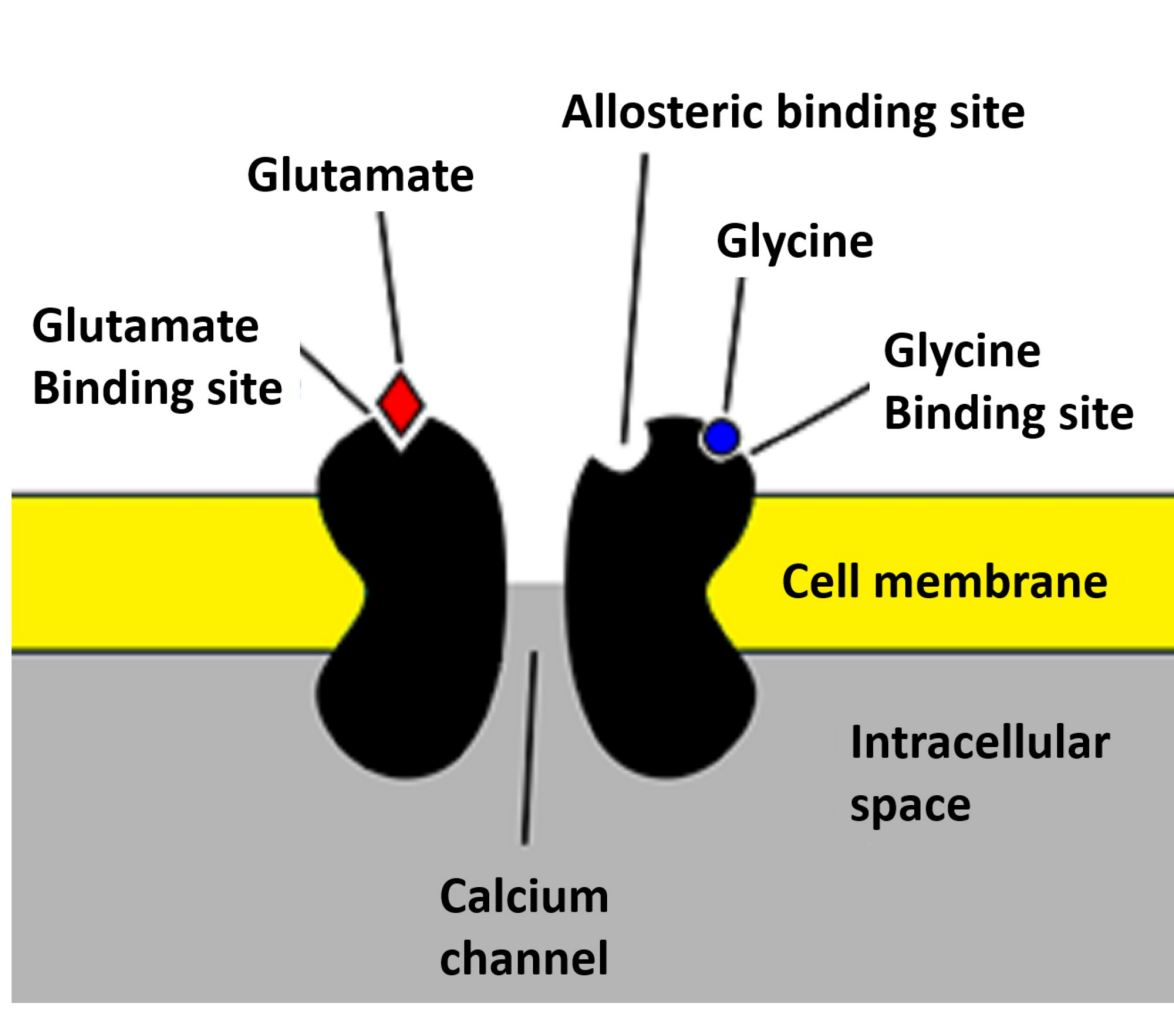
The NMDA receptor Abbreviations: NMDA, N-methyl-D-aspartate.

Glutamate is a major excitatory neurotransmitter in the brain. The NMDA receptor is an ionotropic channel which allows calcium ions into the cell from the extracellular space when activated. The NMDA receptor is composed of a combination of subunits of NR1, NR2 (NR2A–NR2D) and NR3 (NR3A and NR3B). NR1 and NR2 form the two NMDA receptor binding sites for neurotransmitters, where the NR1 subunit binds to glycine and the NR2 subunit binds to glutamate [18] (Figure 2). NR2A activation leads to increased long-term potentiation (LTP), and activation of NR2B leads to a decrease in long-term depression (LTD) [19,20]. Ketamine is a non-competitive NMDA antagonist that inhibits the NMDA receptor by blocking the channels and decreasing their opening frequency [19]. The result is inhibition of inhibitory interneurons, which leads to boosted excitation by raising glutamate levels in the interneurons of both the prefrontal cortex and the hippocampus. At a functional level, this improves the patient’s depressive symptoms.

Ketamine action on neuronal synaptoplasticity

**Figure 3:**
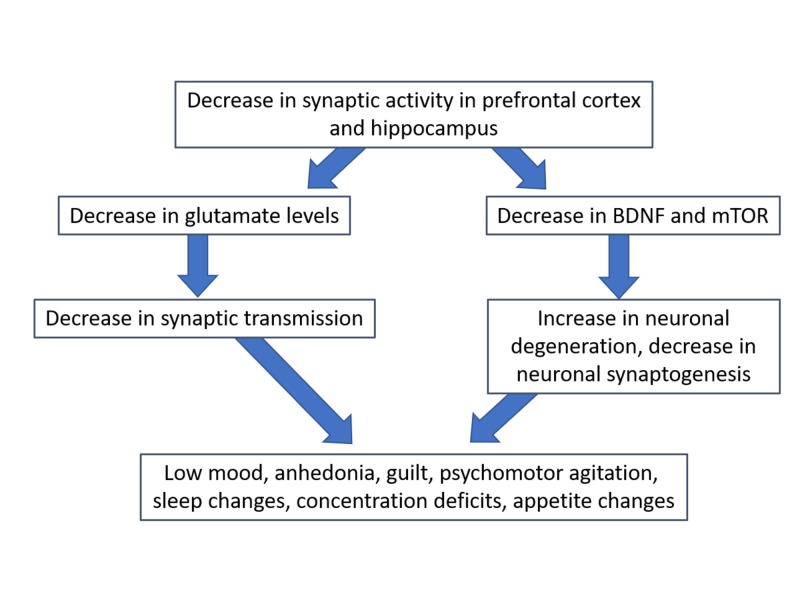
Proposed neurophysiology of treatment-resistant depression Abbreviations: BDNF, brain-derived neurotrophic factor; mTOR, mechanistic target of rapamycin.

Depression is associated with a decreased number of synaptic connections between the prefrontal cortex and hippocampus (Figure 3) [20]. Depressed patients have decreased neuronal synaptoplasticity and LTP in the prefrontal cortex and hippocampus, resulting in disconnection [21,22]. Research on rodents and post-mortem tissues of subjects with depression shows that depression and stress can cause prefrontal cortex pyramidal neuronal atrophy [23]. Neuronal atrophy is accompanied by a decrease in the number of dendritic spines and other alterations in the morphology of dendrites [24-26]. Chronic unpredictable stress (CUS) leads to reduced amplitude and frequency of both 5-HT and hypocretin-induced excitatory postsynaptic potentials (EPSPs) [23]. Decreased 5-HT and hypocretin-induced excitatory postsynaptic currents (EPSCs) affect the synaptic currents in both the cortical-cortical (5-HT) and thalamocortical pathways. The idea that CUS can affect these regions is consistent with studies performed on rats showing CUS causes depression-like behavior, including anhedonic and anxiogenic behaviors [27,28]. Several other studies demonstrated chronic CUS causes a decrease in synaptic proteins, leading to atrophy of neural dendrites in the limbic brain regions including the prefrontal cortex [29-33].

Clinically, increasing the LTP is used to treat a variety of psychiatric illnesses, including depression [34]. Long-term ketamine use can result in changes in neurotrophic factors leading to increased LTP, thus increasing neuronal synaptoplasticity and improving baseline clinical depressive symptoms. Along with ketamine’s proposed effect on synaptoplasticity, it may also act through modulation of intracellular neurotrophic factors, such as BDNF and mTOR pathways.

Ketamine and BDNF

Low levels of BDNF may predispose to neuronal degeneration, neuronal atrophy and decreased dendritic numbers, thus leading to low synaptic activity and clinical symptoms of depression. Multiple studies report depressed patients have low levels of BDNF in the prefrontal cortex and hippocampus. A clinical trial reported 15 of 22 patients who received ketamine showed an increase in BDNF levels at 240 minutes post infusion. The study also reported that a rise in BDNF after ketamine infusion was correlated with a decrease in the Montgomery-Asberg Depression Rating Scale score [35-37]. Increasing BDNF levels may be a significant mechanism of ketamine’s effect on depression since another clinical trial showed direct infusion of BDNF also helped ameliorate depressive symptoms [38].

In the hippocampus, NMDA antagonism by ketamine results in increased levels of BDNF via two main pathways. The first pathway to increased translation of BDNF results from dephosphorylation of eukaryotic elongation factor 2 (eEF2). Ketamine inhibits NMDA receptor-mediated spontaneous miniature excitatory postsynaptic currents (NMDA-mEPSCs), causing a decrease in eEF2 kinase activity, a subsequent decrease in eEF2 phosphorylation, and an increase in dephosphorylation [39]. As a result, there is a rapid increase in the translation of BDNF. The second ketamine NMDA antagonism pathway that increases BDNF levels is phosphorylation of adenosine monophosphate-activated protein kinase, leading to partial BDNF upregulation [40]. The increased release of BDNF from postsynaptic neurons and dendrites enhances presynaptic function, causing homeostatic plasticity [41,42].

Ketamine and mTOR

mTOR is a critical hub of cellular growth and proliferation in neuronal synaptic activity. Recent studies show mTOR signaling in the prefrontal cortex is compromised in depressed patients. Depressed patients have low activity of mTOR and a decreased stimulation of neurons, causing increased depressive symptoms [43]. One of the proposed mechanisms of the rapid antidepressant effects of ketamine is through alteration of the mTOR pathway in the prefrontal cortex, ultimately increasing intracellular protein synthesis. In the prefrontal cortex, antagonism of NMDA receptors by ketamine predominantly affects inhibitory interneurons and causes an increase in alpha-amino-3-hydroxy-5-methyl-4-isoxazole propionic acid receptor-mediated neurotransmission, activating downstream pathways involving mTOR and BDNF and leading to increased synaptic transmission. Ketamine rapidly activates the mTOR pathway and causes an increase in synaptic signaling proteins. It also increases new spine synapses (synapse-associated protein synthesis) and enhances its function and connectivity in the prefrontal cortex, thereby reversing the deficits caused by depression or stress [12]. Some studies on rodents showed antidepressant-like effects and synaptogenesis induced by ketamine when pretreated by rapamycin (mTOR inhibitor) [44,45].

When ketamine binds to the NMDA receptor, it activates and upregulates mTOR signaling, causing phosphorylation of protein kinase B (serine/threonine protein kinase) and extracellular kinases (ERK1 and ERK2), resulting in their activation. The mTOR then targets the translation components, including the eukaryotic initiation factors elF4B, elF4G, and elF4E, and especially the ribosomal recruitment to mRNA [12]. As the translation machinery is the target, ketamine causes an increase in BDNF translation and more protein synthesis in the neuronal synapses of both the hippocampus and prefrontal cortex. This increase in BDNF and other proteins helps in the formation of new spine synapses and their maturation and function [8,46,47]. Among several targets controlled by mTOR, one of the main targets for the antidepressant effect is p70-kDa phosphorylation, a ribosomal protein S6 Kinase. Activated p70S6K helps to initiate protein translation through phosphorylation of eukaryotic initiation factor 4B (eIF4B) [48]. eIF4B activation ultimately results in efficient translation of synaptic proteins such as synapsin I that have a central role in increasing synaptic activity in the prefrontal cortex and ultimately alleviating the acute depressive symptoms [47,49,50].

## Conclusions

Ketamine shows potential as a robust antidepressant in treatment-resistant depression. As an NMDA antagonist, it increases synaptic transmission by increasing glutamate levels and neurotrophic factors that boost synaptic growth. Higher levels of BDNF induce the growth of neuronal dendrites and enhance synaptoplasticity and long-term potentiation, ultimately resulting in the improvement of depressive symptoms. This novel approach to treating depression warrants significantly more research and has promising potential as a future treatment of depression.
